# HER2 isoforms co-expression differently tunes mammary tumor phenotypes affecting onset, vasculature and therapeutic response

**DOI:** 10.18632/oncotarget.17088

**Published:** 2017-04-13

**Authors:** Arianna Palladini, Giordano Nicoletti, Alessia Lamolinara, Massimiliano Dall’Ora, Tania Balboni, Marianna L. Ianzano, Roberta Laranga, Lorena Landuzzi, Veronica Giusti, Claudio Ceccarelli, Donatella Santini, Mario Taffurelli, Enrico Di Oto, Sofia Asioli, Augusto Amici, Serenella M. Pupa, Carla De Giovanni, Elda Tagliabue, Manuela Iezzi, Patrizia Nanni, Pier-Luigi Lollini

**Affiliations:** ^1^ Department of Experimental, Diagnostic and Specialty Medicine, University of Bologna, Bologna, Italy; ^2^ Rizzoli Orthopedic Institute, Laboratory of Experimental Oncology, Bologna, Italy; ^3^ Aging Research Centre, “Gabriele d’Annunzio” University, Chieti, Italy; ^4^ Pathology Unit, Policlinico S.Orsola-Malpighi University Hospital, Bologna, Italy; ^5^ Department of Medical and Surgical Sciences of Bologna, Bologna, Italy; ^6^ Anatomic Pathology, Department of Biomedical and Neuromotor Sciences, Bellaria Hospital, University of Bologna, Bologna, Italy; ^7^ University of Camerino, Camerino, Italy; ^8^ Istituto Nazionale Tumori, Milano, Italy

**Keywords:** HER2, Delta16, trastuzumab, breast cancer, model of host-tumor interactions

## Abstract

Full-length HER2 oncoprotein and splice variant Delta16 are co-expressed in human breast cancer. We studied their interaction in hybrid transgenic mice bearing human full-length HER2 and Delta16 (F1 HER2/Delta16) in comparison to parental HER2 and Delta16 transgenic mice. Mammary carcinomas onset was faster in F1 HER2/Delta16 and Delta16 than in HER2 mice, however tumor growth was slower, and metastatic spread was comparable in all transgenic mice. Full-length HER2 tumors contained few large vessels or vascular *lacunae*, whereas Delta16 tumors presented a more regular vascularization with numerous endothelium-lined small vessels. Delta16-expressing tumors showed a higher accumulation of *i.v*. injected doxorubicin than tumors expressing full-length HER2. F1 HER2/Delta16 tumors with high full-length HER2 expression made few large vessels, whereas tumors with low full-length HER2 and high Delta16 contained numerous small vessels and expressed higher levels of VEGF and VEGFR2. Trastuzumab strongly inhibited tumor onset in F1 HER2/Delta16 and Delta16 mice, but not in full-length HER2 mice. Addiction of F1 tumors to Delta16 was also shown by long-term stability of Delta16 levels during serial transplants, in contrast full-length HER2 levels underwent wide fluctuations. In conclusion, full-length HER2 leads to a faster tumor growth and to an irregular vascularization, whereas Delta16 leads to a faster tumor onset, with more regular vessels, which in turn could better transport cytotoxic drugs within the tumor, and to a higher sensitivity to targeted therapeutic agents. F1 HER2/Delta16 mice are a new immunocompetent mouse model, complementary to patient-derived xenografts, for studies of mammary carcinoma onset, prevention and therapy.

## INTRODUCTION

The HER2 oncogene encodes a tyrosine kinase involved in the onset and progression of breast cancer and other human tumors [1–4]. Current diagnostic and therapeutic criteria are based on the analysis of gene amplification and/or protein expression as if HER2 were translated into a single protein species. On the contrary, tumor cells may contain at the same time different HER2 proteins resulting from mutation, alternative splicing, alternative initiation of translation and post-translational modification [4–15].

Some HER2 isoforms are definitely more oncogenic than full-length HER2 [8, 9, 16], whereas others inhibit carcinogenesis [12]. The Delta16 splice variant, lacking exon 16, has the properties of an activated oncogene [5–7, 10, 17–20] and its expression was recently found to be associated with high stemness and epithelial mesenchymal transition [20, 21]. On the other hand, Delta16 could also play beneficial roles in the response to targeted therapeutic agents, such as the monoclonal antibody trastuzumab [18, 19].

Human breast cancers co-express Delta16 and full-length HER2. Transgenic mice for each isoform (Delta16 or full-length human HER2) have been developed [17, 22], but co-expressing murine models were not studied so far. To study mammary carcinogenesis in a mouse model that mimics the human situation, we produced hybrid mice bearing heterozygous copies of both human transgenes (F1 HER2/Delta16 mice), and we compared them to parental mice (referred to as HER2 and Delta16 transgenic mice, respectively).

## RESULTS

### Mammary carcinogenesis

**Figure 1:**
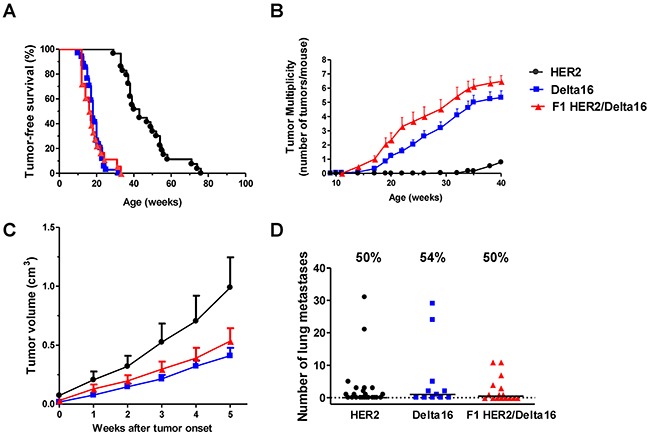
Spontaneous mammary carcinogenesis in F1 HER2/Delta16 mice compared to parental HER2 and Delta16 mice **(A)** Kaplan-Meier tumor-free survival curves. HER2 mice n=29, Delta16 mice n=34, F1 HER2/Delta16 mice n=18. Tumor-free survival of HER2 mice was significantly longer (p<0.001 by the Mantel-Haenszel's test) than that of Delta16 and F1 HER2/Delta16 mice. **(B)** Tumor multiplicity. Each point represents the mean number of tumors/mouse and SEM. HER2 mice n=29, Delta16 mice n=21, F1 HER2/Delta16 mice n=17. By the Student's *t* test (p<0.001), tumor multiplicity of Delta16 mice and F1 HER2/Delta16 mice was significantly higher than that of HER2 mice. **(C)** Kinetics of the growth of the first tumor. After 5 weeks, the average tumor dimension of the groups cannot be compared, because some mice with larger tumors were already euthanized, hence this would have introduced a bias for any further time point. Each point is the mean tumor volume and SEM. HER2 tumors n=25, Delta16 tumors n=37, F1 HER2/Delta16 tumors n=23. By the Student's *t* test the volume of HER2 tumors was significantly larger than that of Delta16 tumors (at least p<0.05 at all time points). **(D)** Autochthonous metastatic spread. Each point represents the number of lung metastases in one mouse, the horizontal bar represents the median. The percentage of mice with metastases are reported above each data set. HER2 mice n=23, Delta16 mice n=11, F1 HER2/Delta16 mice n=16. By the non-parametric Wilcoxon test, no statistical difference was found in the metastatic capacity of the three transgenic mouse models.

Mammary carcinogenesis was slow in transgenic mice carrying the full-length human HER2 gene: the earliest mammary carcinomas appeared around 30 weeks of age, tumor incidence reached 100% well beyond one year of age (Figure 1A). In contrast, the Delta16 isoform induced a much faster carcinogenesis, with early tumors appearing around 10 weeks of age and tumor incidence reaching 100% at 32 weeks of age. These data confirm previously reported tumor-free survival data on HER2 and Delta16 mice [19]. The tumor-free survival curve of F1 HER2/Delta16 mice overlapped that of Delta16 mice running in parallel (Figure 1A). To better appreciate the differences in mammary carcinogenesis progression of three models, we analyzed the number of tumors progressively appearing in each mouse in distinct mammary areas (tumor multiplicity). Delta16 and F1 HER2/Delta16 transgenic lines were prone to the onset of multiple primary carcinomas in different mammary glands with a mean of 5 and 6 tumors, respectively. Conversely, at the same age, HER2 mice developed an average of one carcinoma (Figure 1B). These results indicate that F1 HER2/Delta16 mice shared with Delta16 mice a fast mammary carcinogenesis that affected multiple mammary glands due to the dominant role of Delta16 isoform on tumor onset.

### Tumor progression

After tumor onset, mammary carcinomas of HER2 mice grew faster than those caused by Delta16, in fact average volume after 5 weeks of growth was about twice that of Delta16 mice (Figure 1C).

Delta16 mice developed more tumors than HER2 mice (Figure 1B), but these tumors had a smaller volume than HER2 tumors (Figure 1C). Thus, HER2 and Delta16 mice reached an analogous overall tumor burden at the same moment.

Metastatic spread of human HER2 transgenic mammary carcinomas, as previously found in other HER2/neu transgenic mouse lines [23, 24], was mainly confined to the lungs. Lung metastases were found in about 50% of all mice, regardless of the transgene (Figure 1D), with a median time to euthanasia of 10-12 weeks from tumor onset in the three transgenic mouse lines.

### Expression of full-length HER2 and Delta16 in F1 HER2/Delta16 mammary carcinomas

All mammary carcinomas of the three transgenic mice expressed high levels of total human HER2, as evaluated by means of flow cytometry and immunohistochemical analysis (Supplementary Figure 1). Currently available antibodies do not distinguish the two isoforms, thus we used isoform-specific primers to measure by Real-Time PCR the expression of full-length HER2 and Delta16 in preneoplastic mammary glands and in primary mammary carcinomas.

**Figure 2:**
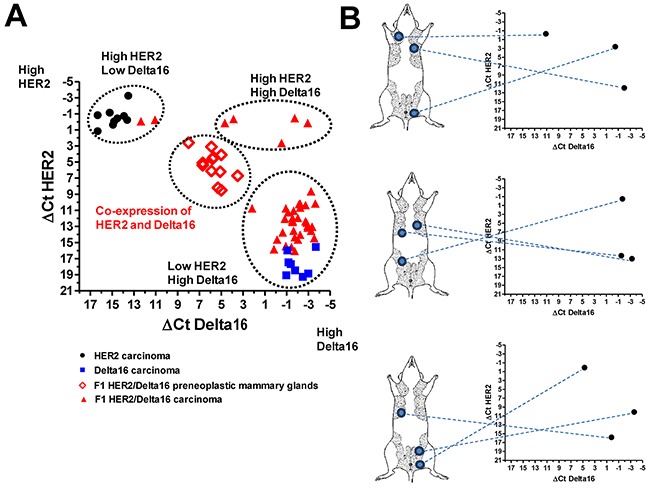
Expression of HER2 and Delta16 transcripts in mammary glands and primary mammary carcinomas ΔCt represents the difference in PCR threshold cycle between the indicated HER2 isoform and reference housekeeping gene GAPDH. **(A)** Each solid point represents one tumor of HER2, Delta16 or F1 HER2/Delta16 mice; each open diamond represents one preneoplastic mammary gland of F1 HER2/Delta16 mice. **(B)** Inter-tumor heterogeneity of HER2 and Delta16 expression among individual mammary carcinomas arising in three mice. For each mouse tumor location is shown on the left and transcript expression in the graph on the right.

Preneoplastic mammary glands of F1 HER2/Delta16 mice co-expressed both isoforms at homogeneous, intermediate levels in comparison to mammary carcinomas of either full-length HER2 or Delta16 transgenic mice. In contrast, mammary carcinomas of F1 HER2/Delta16 mice exhibited three alternative patterns of expression: tumors expressing high levels of both full-length HER2 and Delta16, tumors expressing high levels of full-length HER2 and little, if any, of Delta16 and tumors with a low level of full-length HER2 and a high level of Delta16 (Figure 2A). Thus, neoplastic progression to mammary carcinoma in F1 HER2/Delta16 mice entailed the activation of either or both transgenes. Individual tumors exhibiting different patterns of transgene expression could simultaneously develop within the same host (Figure 2B).

### HER2 and Delta16 expressions tune tumor vascularization

**Figure 3:**
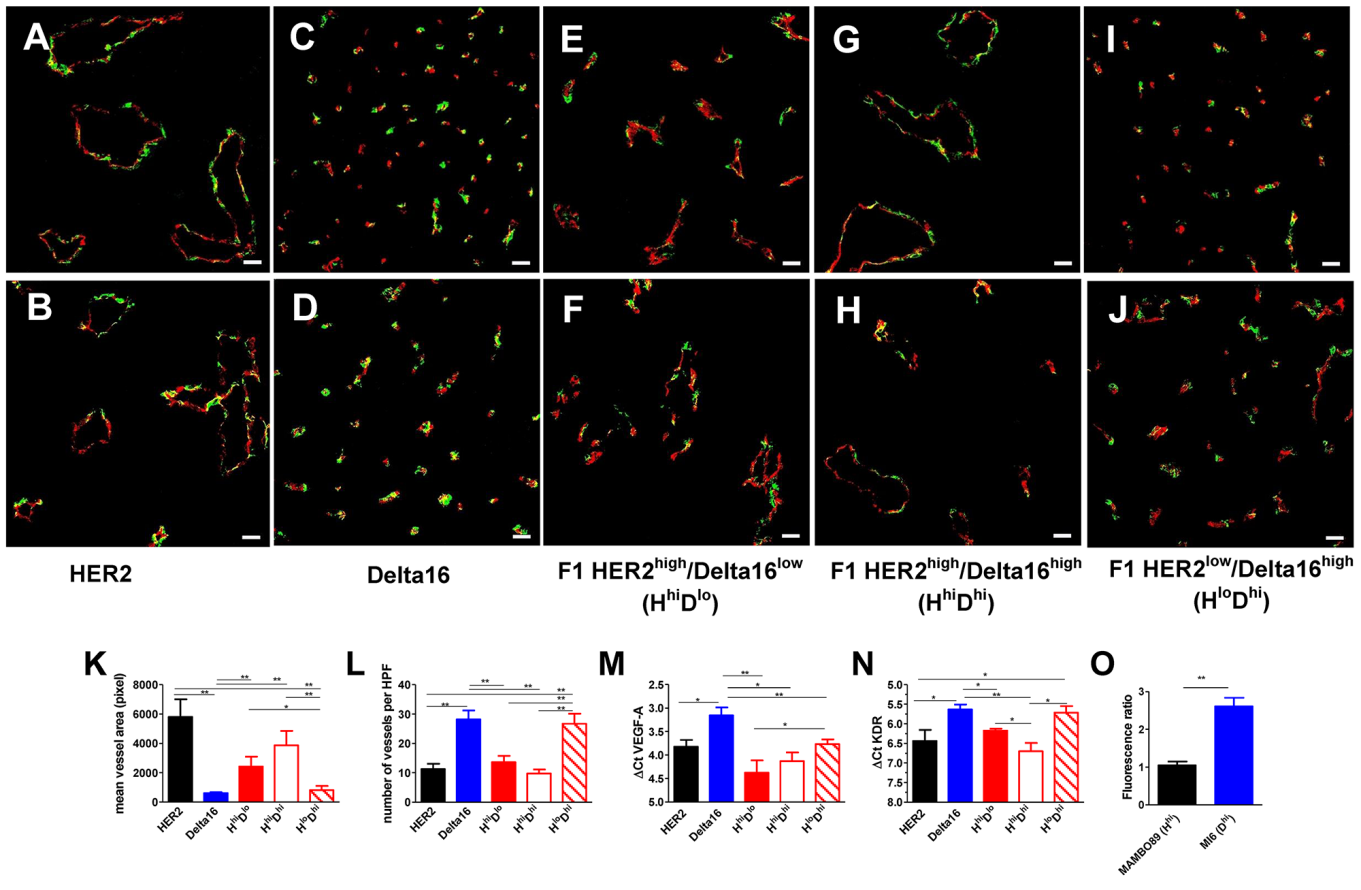
Different angiogenic patterns according to HER2 isoform expression **(A-J)** Representative immunofluorescence images by confocal microscopy of primary mammary carcinomas of the strain indicated below the pictures. CD31-105 (red) and NG2 (green) staining define blood vessels. Scale bar, 50 μm. **(K-L)** Area **(K)** and number **(L)** of vessels per high power field (HPF) x200. Each bar represents the mean and SEM of 5 ×200 fields for each tumor (n=3-5). **(M-N)** Expression of VEGFA **(M)** and KDR **(N)** transcripts. Each bar represents the mean and SEM of 3-19 tumors. ΔCt represents the difference in PCR threshold cycle between the indicated gene and reference housekeeping gene GAPDH. **(O)** Accumulation of doxorubicin in MAMBO89 (HER2^hi^) or MI6 (Delta16^hi^) tumors after the *i.v*. injection of 16 mg/kg of doxorubicin. Fluorescence intensity of doxorubicin in tumor cells was analyzed by flow cytometry and was expressed as fluorescence ratio between tumors of treated and untreated mice. Each bar represents the mean and SEM of 5-6 tumors. *=p<0.05, **=p<0.01 by the Student's *t* test.

A major difference in vascularization between full-length HER2 and Delta16 mammary carcinomas was already evident at necropsy: the former were haemorrhagic, whereas the latter were generally pale. A microscopic study of tumor vascularization showed that full-length HER2 tumors mainly contained few large vessels or vascular *lacunae* (Figure 3A, 3B and 3K and Supplementary Figure 2), whereas Delta16 tumors were perfused by numerous endothelium-lined small vessels (Figure 3C, 3D and 3L and Supplementary Figure 2). Mammary carcinomas of F1 HER2/Delta16 mice presented both types of vascularization (Supplementary Figure 2), however, when we classified the tumors by the prevalent HER2 isoform expressed (*see* Figure 2A), we found that tumors with high full-length HER2 expression showed few large vessels (Figure 3E and 3F), whereas tumors with low full-length HER2 and high Delta16 mainly contained numerous small vessels (Figure 3I and 3J). It should be noted that in F1 HER2/Delta16 tumors expressing high levels of both full-length HER2 and Delta16 (Figure 3G and 3H) the vascular pattern resembled that of full-length HER2. Thus, in the vascular phenotype, full-length HER2 appeared to be dominant.

To investigate the molecular determinants of the different angiogenic patterns, we studied the expression of various genes involved in the control of tumor angiogenesis but no relevant variations (data not shown) were observed for several of these (FGF2, PECAM1, MCAM, COX2). Significant differences, mirroring the angiogenic patterns, were found in the tumor expression of vascular endothelial growth factor (VEGF) and its receptor VEGFR2 (KDR) (Figure 3M and 3N), which were higher in Delta16 and F1 HER2^low^/Delta16^high^ tumors. These results suggest that the angiogenic phenotype characterized by a high number of small vessels, typical of tumors expressing Delta16, was associated with higher cognate expression of VEGFA and of VEGFR2.

Differences in the vascular system could influence drug delivery to the tumor. Thus, we studied doxorubicin accumulation in HER2-expressing tumors (induced by the injection of MAMBO89 cells, established from a mammary carcinoma of a HER2 transgenic mouse) and Delta16-expressing tumors (induced by the injection of MI6 cells, established from a mammary carcinoma of a Delta16 transgenic mouse). Cell lines were used to study a more controlled system, with mice of the same age, carrying a single tumor in each mouse, induced in the same mammary area by the injection of tumor cells with a known expression of HER2 and Delta16. Spontaneous tumors of transgenic mice would have entailed different multiplicities, tumors arising in different mammary areas and at different ages, eventually leading to a more difficult interpretation of doxorubicin accumulation results.

The purpose of this experiment was to evaluate short-term drug accumulation. The level of doxorubicin in tumors, after *i.v*. injection, was evaluated by flow cytometry. Delta16-expressing tumors showed a significant cellular accumulation of doxorubicin as compared to untreated mice, whereas doxorubicin did not accumulate in HER2-expressing tumors (Figure 3O).

### Prevention and growth inhibition of mammary cancer by trastuzumab

We treated F1 HER2/Delta16 mice with trastuzumab to analyze the sensitivity of nascent and established tumors to HER2 targeted therapies.

**Figure 4:**
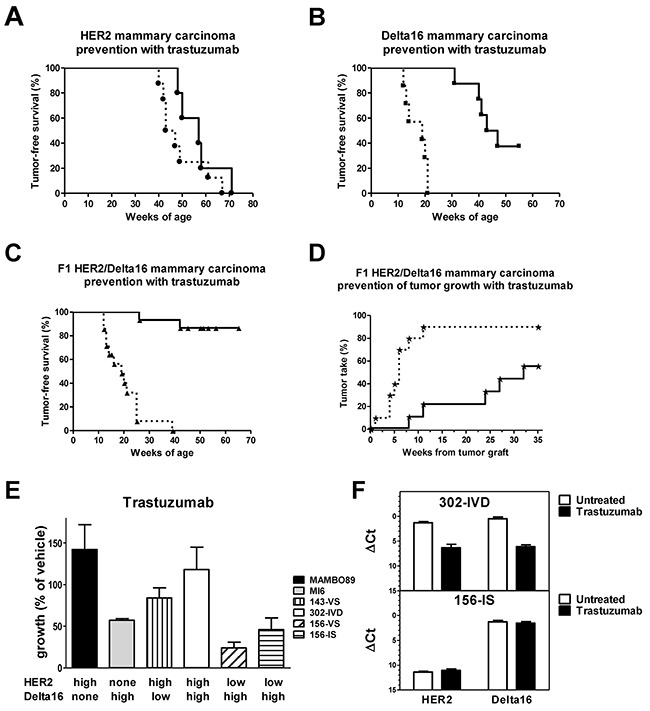
Response to trastuzumab treatment *in vivo* and *in vitro* **(A-C)** Prevention of autochthonous mammary carcinogenesis by trastuzumab treatment. Kaplan-Meier tumor-free survival curves of transgenic mice treated *i.p*. with vehicle (dashed line) or trastuzumab (solid line); statistical comparisons were made by Mantel-Haenszel's test: HER2 mice **(A)** (vehicle n=8, trastuzumab n=5), no significant difference; Delta16 mice **(B)** (vehicle n=7, trastuzumab n=8), p<0.001; F1 HER2/Delta16 mice **(C)** (vehicle n=14, trastuzumab n=15), p<0.001. **(D)** Prevention of growth of F1 HER2/Delta16 tumors implanted in the fourth left mammary fatpad of non-transgenic FVB mice treated with vehicle (dashed line) (n=10) or trastuzumab (solid line) (n=9); p<0.01 by Mantel-Haenszel's test. **(E)** *In vitro* (3D culture) sensitivity to trastuzumab (10 μg/ml) of cell lines derived from HER2, Delta16 and F1 HER2/Delta16 mammary carcinomas. Each bar represents the mean percentage (relative to vehicle-treated cells) and SEM of colonies grown in 2-4 independent experiments. MI6, 156-VS and 156-IS were significantly (p<0.05 by the Student's *t* test) inhibited by trastuzumab. **(F)** Expression of HER2 and Delta16 transcripts in 302-IVD (upper graph) and 156-IS (lower graph) cells after trastuzumab treatment in 3D culture. Each bar represents the mean and SEM of 2-6 independent determinations. ΔCt represents the difference in PCR threshold cycle between the indicated gene and reference housekeeping gene GAPDH. A significant (p<0.01 by the Student's *t* test) inhibition of both transcripts was observed only in 302-IVD cells.

Administration of trastuzumab to full-length HER2 transgenic mice slightly delayed tumor onset (Figure 4A). Conversely, a long delay in mammary carcinogenesis was obtained with trastuzumab in Delta16 mice (Figure 4B). Finally, trastuzumab administration to young F1 HER2/Delta16 mice effectively prevented mammary carcinoma onset (Figure 4C). At one year of age more than 85% of treated mice were tumor-free, whereas none of the untreated mice was tumor-free by 40 weeks of age.

F1 HER2/Delta16 mice are prone to develop multiple primary carcinomas with different HER2 isoform levels (*see* Figure 2A). To investigate the effect of trastuzumab against homogeneous established tumors, we transplanted F1 HER2/Delta16 primary tumors into non-transgenic FVB mice. We used non-transgenic mice to avoid the interference with endogenous mammary carcinogenesis of transgenic mice even if immune response of non-transgenic mice could influence the effect of trastuzumab [25, 26]. A short (one month) treatment with trastuzumab strongly inhibited tumor growth for a long time, with more than 40% mice still tumor-free five months after the end of the treatment (Figure 4D). We analyzed the expression of full-length HER2 and Delta16 after the treatment. No significant difference was found in the isoform levels between trastuzumab treated and untreated tumors (data not shown).

To analyze the intrinsic sensitivity of F1 HER2/Delta16 mammary carcinoma cells to anti-HER2 monoclonal antibody trastuzumab, we established four cell lines representative of three alternative patterns of expression found in F1 HER2/Delta16 tumors (*see* Figure 2A). Cell lines of F1 HER2/Delta16 and parental origin were then exposed to trastuzumab in 3D (agar) cultures. A strong inhibition of cell growth was obtained only when cells expressed high levels of Delta16, either alone (MI6 cells), or together with a low level of full-length HER2 (F1 HER2^lo^/Delta16^hi^cell lines 156-VS and 156-IS). In contrast, all cell lines expressing high levels of full-length HER2 alone (MAMBO89 cells), or together with Delta16, were not inhibited, regardless of the level of Delta16 expression (F1 HER2^hi^/Delta16^lo^ cell line 143-VS and F1 HER2^hi^/Delta16^hi^ cell line 302-IVD) (Figure 4E). These results suggested a dominant role of full-length HER2 in cell autonomous trastuzumab resistance.

Cells from 3D colonies grown after trastuzumab treatment were analyzed to quantify the expression of the two isoforms. 302-IVD cells, resistant to trastuzumab, showed a significant decrease in the level of both isoforms compared to cells grown without antibody. No change was observed in trastuzumab-sensitive 156-IS cells. Finally, trastuzumab treatment did not affect the isoform ratio in both 302-IVD and 156-IS cells (Figure 4F).

### *In vivo* isoform kinetics in murine and human mammary carcinomas

We wanted to verify whether isoform levels were stable *in vivo* in the long term. To this end we studied serial transplants of two F1 HER2/Delta16 mouse tumors in syngeneic immunocompetent hosts (mouse-derived isografts named MoMo1 and MoMo2) in comparison with two patient-derived xenografts (PDX named FO4 and TA18), established in our laboratory from human breast cancers expressing different levels of HER2, growing in immunodeficient NSG mice.

**Figure 5:**
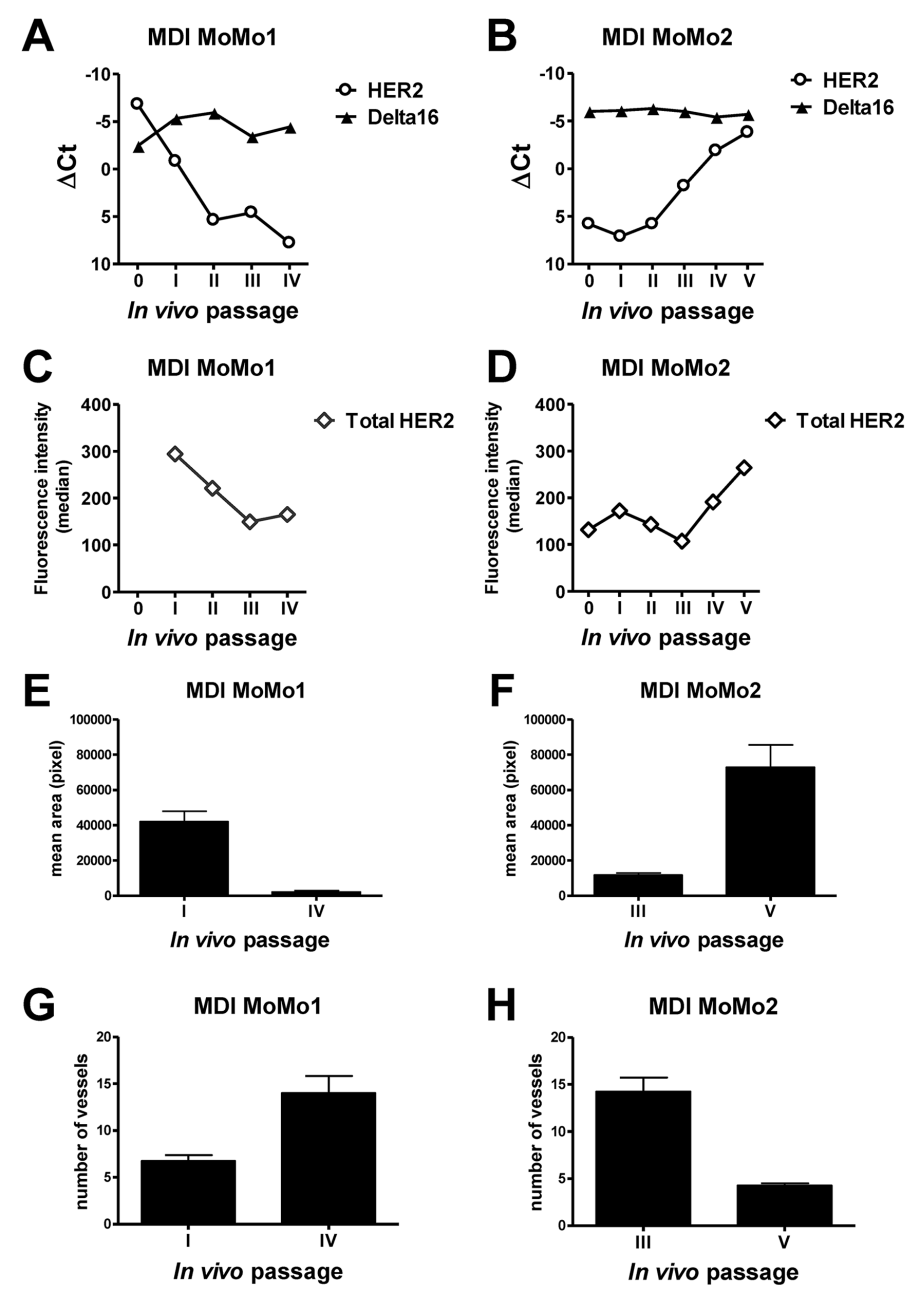
Kinetics of HER2 and Delta16 expression and vascular pattern in serial transplants (mouse-derived isografts, MDI) of F1 HER2/Delta16 mammary carcinomas MDI MoMo1 **(A, C, E, G)** and MDI MoMo2 **(B, D, F, H)**. **A-B**. Expression of HER2 and Delta16 transcripts by Real-Time PCR; ΔCt represents the difference in PCR threshold cycle between the indicated HER2 isoform and reference housekeeping gene mTBP. **C-D**. Total surface HER2 protein by FACS analysis. **E-H** Area **E-F** and number **G-H** of vessels were measured by CD31 immunostaining of tumors; each bar represents the mean and SEM of 4×200 fields for each tumor. Student's *t* test: **(E)** and **(H)**, p<0.001; **(F)** and **(G)**, p<0.01.

Serial *in vivo* passages of MoMo1, with a high expression of both isoforms, and MoMo2, with a higher expression of Delta16 than full-length HER2, were analyzed for each isoform expression by Real-Time PCR (Figure 5A-5B) and for the total protein level by flow cytometry (Figure 5C-5D): Delta16 levels were stable over all passages (which took more than 11 months *in vivo*), whereas the levels of full-length HER2 showed strong variations, including both increases and decreases over time (Figure 5A-5B). As shown for primary tumors (*see* Figure 3), variations in full-length HER2 and Delta16 levels in MoMo1 and MoMo2 were accompanied by changes in vascularization patterns (Figure 5E-5H), thus indicating a functional role of HER2 isoform variations in tumor biology.

**Figure 6:**
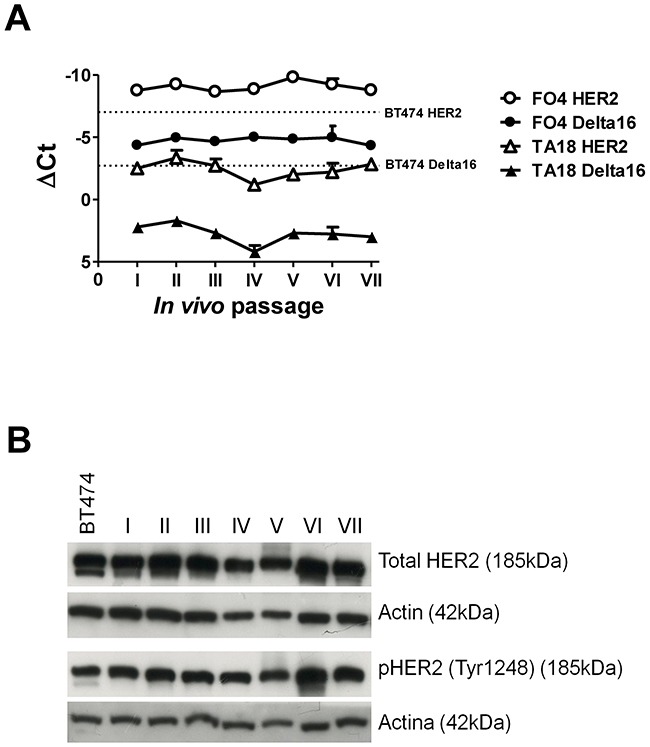
Kinetics of HER2 and Delta16 expression in serial transplants of human breast cancer patient-derived xenografts (PDX) FO4 and TA18 **(A)** Expression of HER2 and Delta16 transcripts by Real-Time PCR; ΔCt represents the difference in PCR threshold cycle between the indicated HER2 isoform and reference housekeeping gene hTBP. For reference, the human HER2 positive cell line BT474 was used (dashed lines) **(B)** Expression of total and phosphorylated HER2 proteins by Western blot in *in vivo* passages of PDX FO4 tumors. For reference, the human HER2 positive cell line BT474 was used.

The same type of kinetic study performed in two PDX showed a remarkable stability of both full-length HER2 and Delta16 isoforms over the course of serial *in vivo* passages (which took more than 15 months *in vivo*) in immunodeficient NSG mice (Figure 6A). Total HER2 protein levels, as measured by Western blot analysis (Figure 6B), were also stable over time.

Fragments of MoMo2 were serially implanted in immunodeficient NSG mice and analyzed during several consecutive *in vivo* passages. The strong fluctuations in HER2 expression observed in immunocompetent mice (Figure 5) were not present in tumors growing in immunodeficent mice, thus suggesting a possible role of the immune system in the modulation of HER2 expression (Supplementary Figure 3).

The level of Delta16 transcript in FO4, MoMo1 and MoMo2 tumors was comparable to that of prototypical human HER2 positive breast cancer cell line BT474 (*see* Figure 5 and Figure 6). Similarly, the level of full-length HER2 in BT474 was close to that of FO4 tumors, MoMo1 original tumor and MoMo2 tumor at the fifth passage. TA18 tumors showed an amount of HER2 and Delta16 transcripts lower than FO4 tumors and BT474 cell line.

## DISCUSSION

One half of all human HER2 positive breast cancers express the Delta16 splicing isoform [10], which is thought to play important roles in determining cancer aggressiveness and response to targeted therapies [10, 17–20]. The first step in HER2 signaling is the dimerization of HER2 molecule on the cell surface. Previous studies have shown that HER2 and Delta16 homo- and hetero-dimers are endowed with different activities, hence isoform interactions should be factored into experimental studies. However, mouse studies so far were performed in transgenics that exclusively express either full-length HER2 or Delta16 [17, 19, 22]. We analyzed mammary carcinogenesis, angiogenesis and therapeutic responses in F1 hybrid mice expressing both human full-length HER2 and Delta16 isoforms, to model isoform interactions at work in human breast cancer.

Mammary carcinogenesis was faster in F1 HER2/Delta16 hybrid mice and a higher number of tumors developed in multiple mammary glands in comparison to mice carrying full-length HER2. The similarity between F1 HER2/Delta16 and Delta16 mice indicates that Delta16 dominates carcinogenesis regardless of the presence of full-length HER2. Delta16 preferentially homodimerizes, and such homodimers are highly active [19], two facts that could explain the dominance of Delta16 in determining tumor onset.

In contrast, after tumor onset, the growth of tumors expressing full-length HER2 was faster than that of Delta16 tumors, possibly as a consequence of microenvironmental interactions of tumor cells. We found striking differences in the vascularization of Delta16 and full-length HER2 tumors. A higher expression of VEGF and its receptor VEGFR2 was found in Delta16 tumors, characterized by a more homogeneous vascular architecture based on numerous small vessels. In contrast, full-length HER2 tumors contained enlarged vessels and vascular *lacunae*, which could favor a faster tumor growth and dissemination. Vascularization of F1 HER2/Delta16 tumors showed both types of patterns found in parentals, proportional to HER2 and Delta16 levels. Concordant association between high HER2 expression and atypical vessels was found in human breast cancer [27, 28]. Our data suggest a dominant role of full-length HER2 as the driver of an abnormal vascularization. Our results warrant a future study of human breast cancer vascularization in relation to HER2 isoform expression.

Previous studies, including our own [10, 18, 19], showed that *in vitro* invasiveness and lung colonization after intravenous injection of cultured cells expressing full-length HER2 were lower than those of Delta16 expressing cells. In addition Turpin and colleagues [20] reported an increased number of lung metastases in an inducible Delta16 transgenic mouse model, with an extremely short latency period, respect to HER2 mice. Here, we found similar numbers of autochthonous lung metastases in all transgenic mice, regardless of the HER2 isoform levels. Although the time from tumor onset to euthanasia was similar for all three strains, the longer natural history of tumors in HER2 mice could increase the probability of metastatic spread in these mice. On the other hand, the higher tumor multiplicity could increase metastatic dissemination in Delta16 and in F1 HER2/Delta16 mice. In addition, whereas previous studies underlined the intrinsic proliferative/invasive disadvantage of full-length HER2 mammary carcinoma cells in comparison to Delta16, our results indicate that microenvironmental interactions, *e.g*. with tumor vasculature [29], could counterbalance this disadvantage by granting preferential access to the bloodstream and to systemic dissemination, eventually resulting in net metastatic diffusion of equal entity. Our conclusion on autochthonous metastases is consistent with clinical data from Mitra and colleagues who reported an increase of lymph nodal invasion, but not of systemic dissemination, in association with Delta16 expression [10]. On the other hand, Alajati and colleagues reported a correlation between a Delta16 gene signature and a poor distant metastasis-free survival [18]. Further studies are needed to solve this issue.

The expression of the Delta16 isoform was initially thought to be involved in resistance to HER2 targeted therapies. These studies were mainly performed *in vitro* with cell lines transduced with HER2 isoforms. However, we have recently shown that Delta16 expression is actually associated with an increased trastuzumab sensitivity of transgenic mouse tumors, and with a decreased risk of relapse after trastuzumab treatment in breast cancer patients [19]. Susceptibility to trastuzumab was also reported for MCF10A-Delta-HER2 cells (expressing Delta16 only) injected into immunodeficient mice [18]. In the present work, trastuzumab treatment prevented the onset of mammary carcinoma, showing a higher efficacy in the prevention of Delta16-expressing tumors with respect to tumors expressing only the full-length HER2 isoform. Therefore, even in tumor prevention, expression of the Delta16 isoform conferred a higher sensitivity to trastuzumab. We found that HER2 expression could also confer a lower susceptibility to cytotoxic drugs through limited drug accumulation due to a more irregular vascularization. Furthermore, *in vitro* we evidenced an intrinsic trastuzumab susceptibility of Delta16-expressing cells, with low or absent HER2. We and other reported a strong activation of Src associated to Delta16 expression [10, 17, 19, 30] and we reported that a high level of Src activation correlated with a better prognosis for patients treated with trastuzumab in adjuvant therapy. Moreover the high level of homodimers in Delta16-expressing cells [17, 19] could explain the strong activity of trastuzumab in presence of Delta16 [31].

The natural history of HER2 tumors can only be inferred by patients’ data. Even if HER2 is a driver of mammary carcinogenesis, its expression was found in ductal carcinoma *in situ* (DCIS), but can decrease in invasive tumors [32–34]. Furthermore, discordance in HER2 expression and drug susceptibility between primary tumors and metastases has been reported [35, 36], thus suggesting variation in HER2 expression during tumor progression. Our results in F1 HER2/Delta16 mice show that the two HER2 isoforms can undergo independent fates. We found a remarkable long-term stability of Delta16 expression during several months of serial transplants of F1 transgenic mammary carcinomas, *vis-à-vis* considerable fluctuations of full-length HER2. These results suggest that Delta16 expression was constrained by its driver role in this tumor system, whereas full-length HER2 could freely fluctuate because it played a lesser role. It should be noted that a measure of the total HER2 protein levels with a monoclonal antibody basically mirrors the variations in full-length HER2, but gives no information on Delta16 expression, thus suggesting that only an assessment of HER2 isoforms could provide a reliable estimate of the functionally relevant oncoproteins in human breast cancer.

At variance with isografts in immunocompetent mice, patient-derived xenografts in immunodeficient mice did not show variations in the levels of HER2 and Delta16 isoforms during serial *in vivo* passages. The presence of a fully-functioning immune system could play a role in determining the variation of the full-length HER2 isoform in immunocompetent mice offering the opportunity to investigate the different immunogenicity of the isoforms. PDX are now proposed as the best model to study new therapies since PDX maintain the main features of a patient's tumor, including the intratumoral heterogeneity. We advocate the need to integrate data from PDX models with the study of genetically-modified immunocompetent tumor models, such as the new model of transgenic F1 mice co-expressing HER2 and Delta16 isoforms described here.

The study of the natural history of tumors in F1 HER2/Delta16 mice showed that, even in genetically identical mice, a divergent evolution can occur, leading to tumors with a variable expression of HER2 isoforms, remindful of the human situation.

In conclusion, we showed that co-expression of HER2 and Delta16 isoforms tunes tumor onset, growth, vascularization and sensitivity to targeted therapy. Our results suggest that prospective clinical studies including a quantitative evaluation of full-length HER2 and Delta16 could improve the prediction of breast cancer sensitivity and resistance to cytotoxic and HER2 targeted therapies.

## MATERIALS AND METHODS

### HER2 transgenic mice and autochthonous mammary carcinomas

Human HER2 transgenic mice [22], here referred to as HER2 mice, were obtained from Genentech (South San Francisco, CA, USA) and bred in our animal facilities. Human Delta16 transgenic mice [17] are here referred to as Delta16 mice. Delta16 male mice and HER2 female mice were crossed to obtain a double transgenic human HER2/Delta16 progeny, here referred to as F1 HER2/Delta16 mice. Female mice were monitored weekly by palpation and tumor dimensions were measured with calipers. Masses with a mean diameter exceeding 3 mm were considered tumors. Mice were euthanized when tumor burden was equivalent to 10% of body mass. Tumor volume was calculated as (π/6)(√*ab*)^3^, where *a* = maximal tumor diameter and *b* = maximal tumor diameter perpendicular to *a*. At necropsy, tumor masses were collected for gene expression and histological analysis. Non-neoplastic mammary glands were collected from 5-week-old F1 HER2/Delta16 mice. Lungs were perfused with black India ink to outline metastases, and fixed in Fekete's solution. Lung metastases were counted under a dissection microscope. All *in vivo* experiments were approved by the institutional review board of the University of Bologna, authorized by the Italian Ministry of Health and done according to Italian and European laws and guidelines.

### Mouse-derived isograft (MDI) model

Fragments of F1 HER2/Delta16 mammary tumors were serially implanted in the fourth left mammary fatpad of 5-17–week–old HER2 transgenic female mice and 5-8-week-old NOD-SCID-Il2rg^−/−^ (NSG) female mice, obtained from The Jackson Laboratories (Sacramento, CA, USA). Tumors grown from serial grafts of fragments from two F1 HER2/Delta16 tumors (here referred to as MoMo1 and MoMo2 respectively) were harvested for molecular investigation and also immediately implanted into other mice.

### Patient-derived xenograft (PDX) model

Two patient-derived xenografts expressing different HER2 levels, named FO4 and TA18, were originally established in our laboratory from human breast cancers by implantation in the fourth left mammary fatpad in 5-10 week-old NSG female mice. The PDXs used in the present work were between the 1^st^ and the 7^th^ serial passage in NSG mice.

### Histological and immunofluorescence analyses of tumor samples

Whole mounts were prepared as previously described [37]. Tumor samples were fixed in 10% neutral buffered formalin and embedded into paraffin or fixed in 4% PFA and frozen in a cryo-embedding medium (OCT, BioOptica, Milan, Italy); 5 μm slides were cut and stained with Hematoxylin (BioOptica) and Eosin (BioOptica) for histological examination. For immunohistochemistry, slides were deparaffinized, serially rehydrated and, after the appropriate antigen retrieval procedure, stained with rabbit polyclonal anti-Human HER2 antibody (A0485, Dako Italia sr.l, Milan, Italy) or with anti-mouse CD31 (DIA-310, Dianova GmbH, Hamburg, Germany), followed by the appropriate secondary antibody (Jackson ImmunoResearch, West Grove, PA, USA). Immunoreactive antigens were detected using streptavidin peroxidase (Thermo Fisher Scientific, Waltham, MA, USA) and the DAB Chromogen System (Dako) or the Vulcan Fast Red Chromogen (Biocare Medical, Concord, CA, USA). After chromogen incubation, slides were counterstained in Hematoxylin (BioOptica) and images were acquired by Leica DMRD optical microscope (Leica, Wetzlar, Germany). For immunofluorescence, 4-6 μm cryostat sections were air-dried, fixed in ice-cold acetone for 10 min and incubated with the following primary antibodies: rat monoclonal anti-CD31 (550274, BD Pharmingen, BD Biosciences, San Jose, CA, USA) mixed with rat monoclonal anti-CD105 (550546, BD Pharmingen) and rabbit polyclonal anti-NG2 (ab5320, EMD Millipore, Billerica, MA, USA) followed by secondary antibodies conjugated with Alexa 546 and Alexa 488 (Thermo Fisher Scientific), respectively. The mixture of antibodies against CD31 and CD105 was used to increase the probability of staining all of the tumor endothelium [38]. The same secondary antibody was used because there was not the need of distinguishing the presence and the quantity of each single protein on cell surface. Image acquisition was performed using Zeiss LSM 510 META confocal microscope. The number and area of vessels were evaluated on the digital images of 3-5 tumors per group (5 fields per tumor at 200x microscope) by 2 pathologists, independently and in a blind fashion. Vessels area (in pixels) was evaluated with Adobe Photoshop by selecting vessels with the lasso tool and reporting the number of pixels indicated in the histogram window.

### Cell lines

Cell line MAMBO89 was previously established from a mammary carcinoma of a HER2 transgenic mouse [39] and in our previous papers referred to as *syn-HER2* [39] or WTHER2 [19]; cell line MI6 was previously obtained from a mammary carcinoma of a Delta16 mouse [19]. We also established four cell lines from F1 HER2/Delta16 mammary carcinomas, named 143-VS, 302-IVD, 156-VS and 156-IS. All cell lines were cultured in Mammocult medium added with the recommended supplements (StemCell Technologies, Vancouver, Canada) + 1% FBS (Thermo Fisher Scientific) and were maintained at 37°C in a humidified 5% CO_2_ atmosphere. All cell lines had been derived in our Laboratories in recent years and are currently monitored for murine origin by PCR, full-length HER2 or Delta16 isoform transgene expression by PCR and flow cytometry and the cell line-specific ratio of the two isoforms by Real-Time PCR.

BT474 breast cancer cell line was authenticated by DNA fingerprinting on the 11^th^ November 2010 (performed by DSMZ, Braunschweig, Germany). Cells were routinely cultured in Roswell Park Memorial Institute (RPMI) medium supplemented with 10% FBS and were maintained at 37°C in a humidified 5% CO_2_ atmosphere. All medium constituents were purchased from Thermo Fisher Scientific.

### RNA extraction and real-time PCR

RNA was extracted from frozen mammary glands, mammary tumors or cells by Trizol reagent (Thermo Fisher Scientific). Gene expression was analyzed by Real-Time PCR using Thermal Cycler CFX96 (Bio-Rad Laboratories, Hercules, CA, USA). Real-Time PCR was performed using Sso Advanced SyBR Green Supermix (Bio-Rad Laboratories) reagents. Evaluated target genes were: human HER2 and Delta16 [10]; mouse VEGF-A (qMmuCID0024411) and mouse VEGFR2 (KDR) (qMmuCID0005890) (Bio-Rad Laboratories). Mouse GAPDH [40], mouse TBP [41] and human TBP [41] were used as endogenous reference genes (indicated as ref gene). Gene expression was evaluated as ΔCt_target gene_ (Ct_targetgene_ – Ct_ref gene_).

### Total HER2 protein detection

Single cell suspensions of tumor masses were analyzed for total HER2 expression on the membrane. Rat anti-mouse CD16/CD32 clone 2.4G2 antibody Fc block (BD PharMingen) was used to block Fc receptors in tumor-infiltrating leukocytes, to avoid the binding of mouse monoclonal antibodies through the Fc fragment instead of through the antigen-binding moiety. Mouse anti-human HER2 primary antibody, clone MGR-2, (Enzo Life Science, Farmingdale, NY, USA) and Alexa Fluor® 488-conjugated goat anti-mouse IgGs (H+L) secondary antibody (Thermo Fisher Scientific) were used. Fluorescence intensity was determined through flow cytometry (FACScan, Becton Dickinson), analysis was performed with FCS EXPRESS 4 (De Novo Software, Glendale, CA, USA).

### Western blot analysis

Frozen tumor tissue was completely immersed in lysis buffer consisting of Novagen PhosphoSafe Extraction Reagent (EMD Millipore) plus phosphatase and protease inhibitors (Sigma-Aldrich, St Louis, MO, USA). Tissue was dissociated and homogenized by gentleMACS Octo Dissociator (Miltenyi Biotech GmbH, Bergisch Gladbach, Germany) and then incubated for 10 minutes at room temperature. Nuclei were removed by centrifugation at 12,000 RCF at 4°C for 15 minutes, and protein concentration in the supernatants was determined by DC Protein Assay (Bio-Rad Laboratories) using bovine serum albumin as standard. Proteins were separated on an 8% polyacrylamide gel (20 μg of total lysate) and then transferred to polyvinylidene difluoride membranes (Bio-Rad Laboratories). After blocking with PBS containing 0.1% Tween 20 plus 5% nonfat dry milk for 1 hour at room temperature, membranes were incubated overnight at 4°C with primary antibodies diluted in blocking buffer. Anti-c-ErbB2/c-Neu (Ab3) mouse monoclonal antibody (3B5) (1:1000; Calbiochem/EMD Chemicals, San Diego, CA, USA), anti-p-Neu (Tyr 1248)-R rabbit polyclonal antibody (sc-12352-R; 1:1000; Santa Cruz Biotechnology, Santa Cruz, CA, USA) and anti-actin rabbit antibody (1:800; Sigma-Aldrich) were used as primary antibodies. After incubation with the respective horseradish peroxidase–labeled secondary antibodies (Santa Cruz Biotechnology), protein presence was revealed by chemiluminescence reaction (LiteAblotplus chemiluminescence substrate; EuroClone, Milan, Italy).

### Treatments with trastuzumab *in vitro* and *in vivo*

*In vitro* sensitivity to trastuzumab (10 μg/ml) was evaluated in three-dimensional cultures (0.33% soft agar containing the drug). Cells were seeded at 10,000 cells/well in 24-well plates. Colonies were counted two to three weeks after seeding. Cells from grown colonies were then collected and seeded in adherent culture for subsequent molecular analyses. *In vivo*, to study the prevention of primary tumor growth, HER2 mice, Delta16 mice and F1 HER2/Delta16 mice were treated twice weekly intraperitoneally (*i.p*.) with trastuzumab (4 mg/kg) starting from 17 weeks of age for HER2 mice, and from 5-8 weeks of age for Delta16 and F1 HER2/Delta16 mice. The start of the treatment was chosen in relation to the tumor latency of each transgenic mouse line. The analysis of whole mounts showed that at the start of treatments the mammary glands did not contain tumors (Supplementary Figure 4). Control mice were treated with physiological solution. To study the inhibition of tumor growth, fragments of a F1 HER2/Delta16 tumor pool (ΔCt HER2, 8.89; ΔCt Delta16 -0.88. Endogenous reference gene: GAPDH) were implanted in the fourth left mammary fat pad of 10-16-week-old FVB (non-transgenic) female mice (FVB/NCrl, purchased from Charles River Laboratories, Calco, Como, Italy). Starting 7 days after fragments implantation, when tumors were not yet palpable, mice received twice weekly the intraperitoneal injection of trastuzumab (4 mg/kg) for four weeks; control mice received physiological solution. Mice in each litter were randomly assigned to treatments one week before the start of treatments.

### Doxorubicin accumulation *in vivo*

FVB mice bearing tumors induced by the injection of MAMBO89 or MI6 cell lines received intravenous injection of 16 mg/kg doxorubicin. Mice were euthanized 2 hr after doxorubicin injection and tumors were dissociated mechanically and enzymatically. Tumor cell suspensions were then analyzed by flow cytometry for doxorubicin fluorescence.

### Statistical analysis

Tumor-free survival curves were compared using the Mantel-Haenszel test. All other comparisons were performed with the Student's *t* test and Wilcoxon and Mann-Whitney non-parametric tests.

## SUPPLEMENTARY MATERIALS FIGURES


