# Supplementary material for: HER2 isoforms co-expression differently tunes mammary tumor phenotypes affecting onset, vasculature and therapeutic response
Source: Oncotarget. 2017 Apr 13;8(33):54444–58. doi: 10.18632/oncotarget.17088 (PMC5589593; doi:10.18632/oncotarget.17088)
Supplement: Supplementary file 1 [file oncotarget-08-54444-s001.pdf]

HER2 isoforms co-expression differently tunes mammary tumor phenotypes affecting onset, vasculature and therapeutic response

SUPPLEMENTARY FIGURES

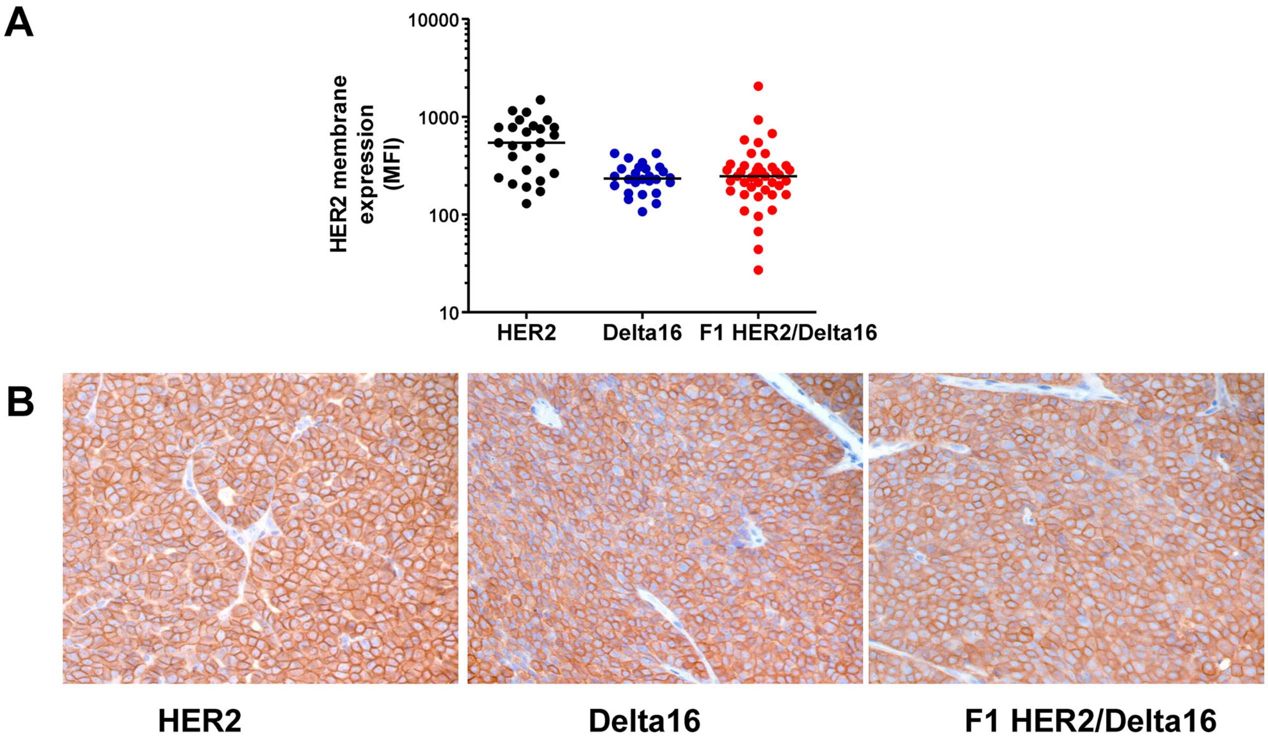

Supplementary Figure 1: Expression of total HER2 protein in primary transgenic mammary carcinomas. (A) Surface HER2 level quantified by flow cytometry. Each point represents the median fluorescence intensity (MFI) of dissociated cells from one tumor; horizontal bars represent the median value of all tumors. HER2 vs Delta16 $p<0.001$, HER2 vs F1 HER2/Delta16 $p<0.001$ by the Mann-Whitney test. **(B)** Representative HER2 immuno-staining images of primary mammary carcinomas of the indicated mice (x400).

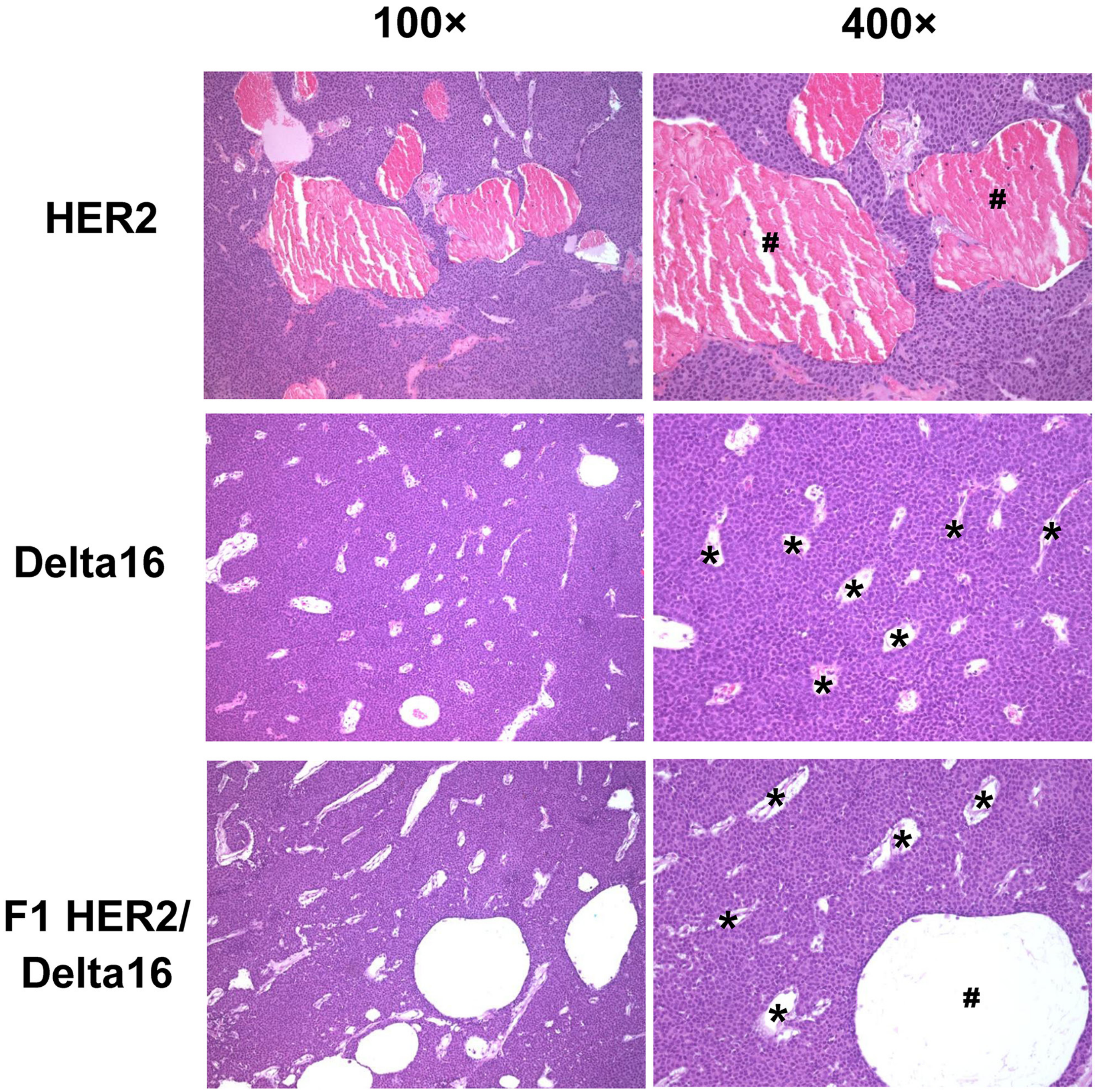

Supplementary Figure 2: Different vascular patterns in transgenic mammary carcinomas of each line. Few large vessels or vascular *lacunae* (#) in full-length HER2 tumors, numerous endothelium-lined small vessels (*) in Delta16 tumors and both types of vascularization in F1 HER2/Delta16 tumors. Hematoxylin-eosin staining (left column: ×100; right column: ×400) are shown.

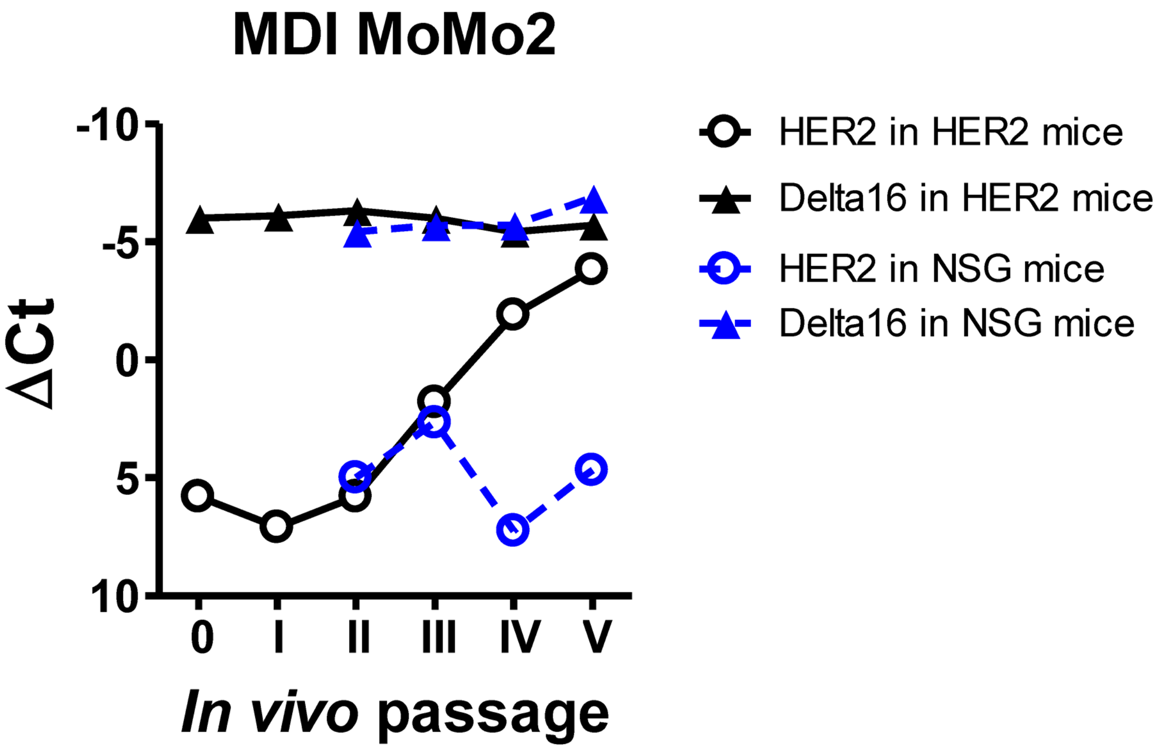

Supplementary Figure 3: Kinetics of HER2 and Delta16 expression of MoMo2 in HER2 and NSG mice. Expression of HER2 and Delta16 transcripts by Real-Time PCR; ΔCt represents the difference in PCR threshold cycle between the indicated HER2 isoform and reference housekeeping gene mTBP.

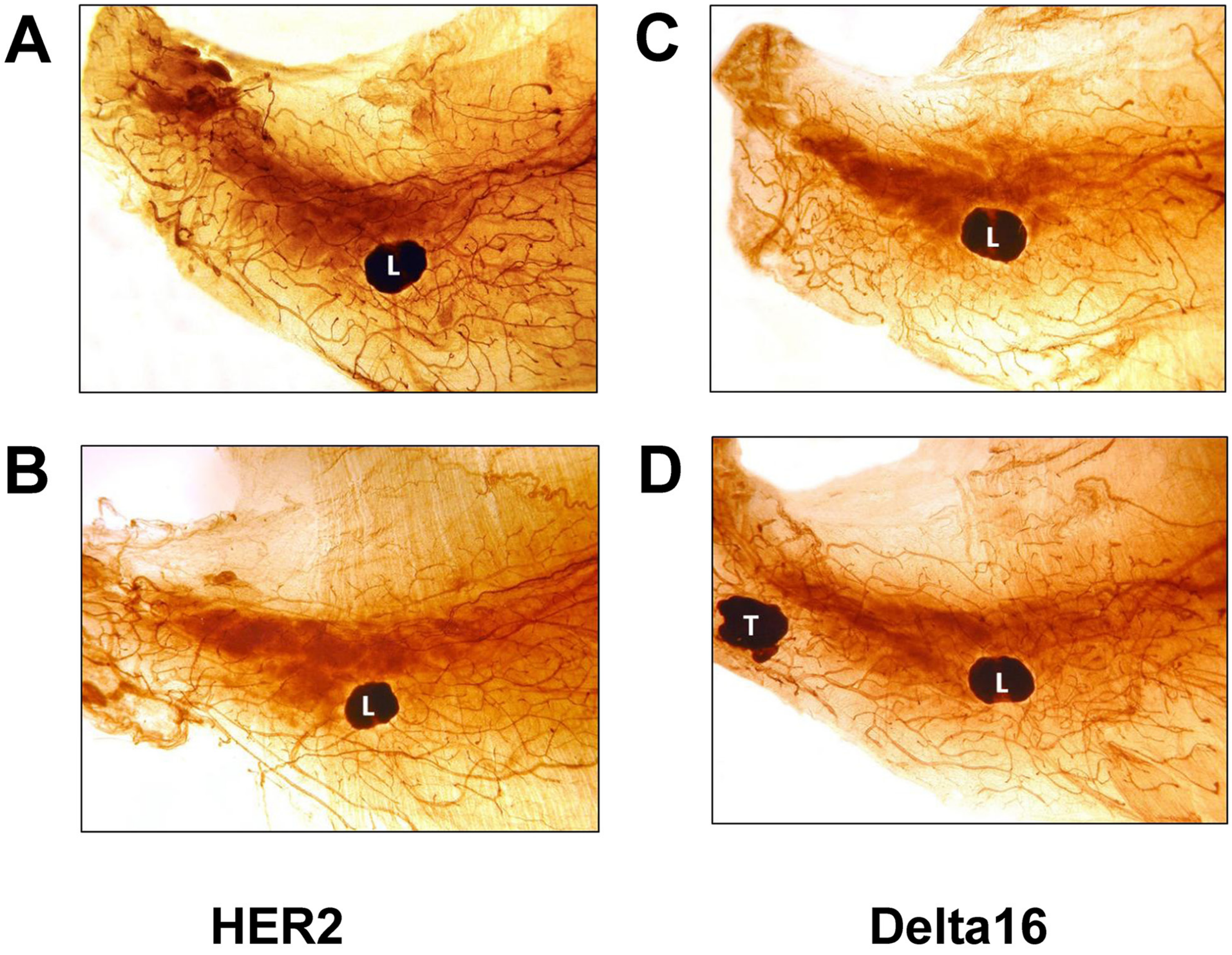

Supplementary Figure 4: Whole mount preparations from representative inguinal mammary glands of HER2 and Delta16 mice. **(A-B)** HER2 mice mammary glands were obtained from 14-week-old **(A)** and 25-week-old **(B)** mice. **(C-D)** Delta16 mammary glands were obtained from 8-week-old **(C)** and 13-week-old **(D)** mice. T, tumor mass; L, inguinal lymph node.
